# lncRNA/MicroRNA interactions in the vasculature

**DOI:** 10.1002/cpt.355

**Published:** 2016-03-31

**Authors:** MD Ballantyne, RA McDonald, AH Baker

**Affiliations:** ^1^ Centre for Cardiovascular ScienceUniversity of Edinburgh, Queen's Medical Research InstituteEdinburghUK; ^2^ Institute of Cardiovascular and Medical ScienceBritish Heart Foundation GlasgowGlasgowUK

## Abstract

MicroRNA (miRNA) have gained widespread attention for their role in diverse vascular processes including angiogenesis, apoptosis, proliferation, and migration. Despite great understanding of miRNA expression and function, knowledge of long noncoding RNA (lncRNA) molecular mechanisms still remains limited. The influence of miRNA on lncRNA function, and the converse, is now beginning to emerge. lncRNA may regulate miRNA function by acting as endogenous sponges to regulate gene expression and miRNA have been shown to bind and regulate lncRNA stability. A detailed understanding of the molecular and cellular effects of lncRNA‐miRNA‐mediated interactions in vascular pathophysiology could pave the way for new diagnostic markers and therapeutic approaches, but first there is a requirement for a more detailed understanding of the impact of such regulatory networks.

It is well established that vascular diseases such as atherosclerosis and ischemic heart disease are among the leading causes of healthcare burden, morbidity, and mortality in Western society.^1^ Endothelial injury, a phenotypic alteration in endothelial function, appears to be one of the initiating factors of vascular disease. Pathological changes are accompanied by proinflammatory cytokines and growth factors and reduction in antithrombogenic agents, leading to a reduction in endothelial integrity. Together the diverse range of cytokines and growth factors are believed to initiate smooth muscle cell proliferation and migration leading to vessel disease and occlusion. Very few systemic therapies exist to treat vascular stenosis, thus new therapies are urgently required. One such mechanism may be through the utilization of noncoding RNA. Both microRNA (miRNA) and long noncoding RNA (lncRNA) have been implicated in pathological processes involved in vascular disease. Interestingly, direct binding of lncRNA and miRNA and subsequent alteration in the function of these molecules has now been discovered.^2^ Here we review the abnormalities in cellular functioning of vascular endothelial and smooth muscle cells, key interactions between novel miRNA and lncRNA, and how these interactions may be utilized therapeutically.

## VASCULAR ENDOTHELIAL CELLS

**Figure 1:**
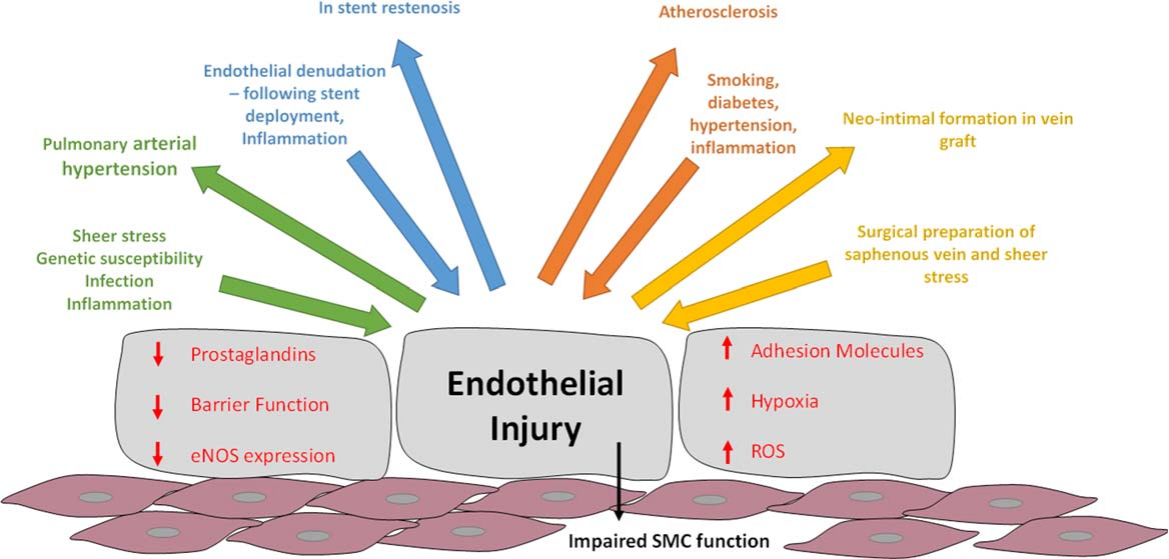
Vascular injury initiating factors. Vascular injury is a multicomponent condition resulting from a plethora of initiating factors, such as sheer stress, endothelial denudation caused by stent deployment, and smoke toxins. This leads to alterations in endothelial function such as a reduction in antithrombotic agents such as nitric oxide (NO) and prostaglandins, initiation of inflammatory signaling cascades, upregulation of adhesion molecules, an increase in reactive oxygen species (ROS), and decreased barrier function. Downward arrows indicate the initiating factors causing endothelial injury while the upward arrows indicate the vascular disease or complication resulting from the initiating factor. Ultimately, changes in endothelial function and release of inflammatory stimuli and growth factors from inflammatory cells impairs the function of the underlying vascular smooth muscle cells causing increased proliferation and migration in response to injury.

The vascular endothelium is no longer thought of as an inert barrier, but a critical regulator of vascular homeostasis. It is widely accepted that endothelial cells maintain vascular integrity and prevent thrombosis following the secretion of a number of antithrombotic mediators, such as prostaglandins and nitric oxide (NO),^3^, ^4^ that prevent platelet activation and inflammatory cell infiltration. Although endothelial activation and initiation of the thrombotic cascade plays a prominent role in wound healing, prolonged periods of endothelial dysfunction and activation can lead to the initiation and progression of a number of vascular pathologies, such as in stent restenosis and intimal formation in response to vein grafting. A number of studies have demonstrated that acute injury or exposure to oxidized lipids can induce activation of the inflammatory nuclear transcription factor κB (NF‐κB), resulting in increased expression of inflammatory cytokines such as interleukin (IL‐1), tumor necrosis factor alpha (TNF‐α), and monocyte chemoattractant protein‐1 (MCP‐1), which upregulate a number of adhesion molecules that enhance leukocyte recruitment and adhesion to the arterial wall (reviewed in more detail by Libby *et al*.^5^). Indeed, a number of studies in genetically modified mice have demonstrated that loss of TNF‐α or IL‐1 or their receptors substantially reduces neointimal formation following ligation injury.^6^, ^7^ In concordance, IL‐1 receptor antagonist (IL‐1Rα)‐deficient mice develop increased neointimal formation in response to injury,^8^ whereas IL‐1Rα administration to mice maintained on an atherogenic background reduces fatty lesion formation.^9^ **Figure** 1 highlights the key initiating steps in endothelial dysfunction for several vascular pathologies. Endothelial denudation and medial wall injury are the initial effects caused by balloon and/or stent deployment following percutaneous coronary intervention (PCI). Increased sheer stress and genetic susceptibility promotes endothelial injury in the setting of pulmonary arterial hypertension (PAH), while well‐known cardiovascular risk factors initiate the vascular injury and “repair process” leading to atherosclerosis.

## ROLE OF SMOOTH MUSCLE CELLS AND GROWTH FACTORS

The fully differentiated vascular smooth muscle cells (VSMC) of human vessels are typically quiescent and are characterized by a low proliferative index. However, unlike many mature cells in the adult body, they do not terminally differentiate and retain remarkable plasticity. They have the ability to switch between a differentiated and quiescent contractile state and a highly migratory and proliferative synthetic state.^10^ This ultimately requires activation of “defined” gene networks (reviewed in detail in a recent review by McDonald *et al*.^11^).

In addition to inflammatory cytokines, a series of eminent studies by Reidy and co‐workers determined that a number of growth factors, basic fibroblast growth factor (bFGF) and platelet‐derived growth factor (PDGF), are potent mitogens and chemoattractants that stimulate neointimal formation following injury. In brief, in the rat carotid balloon injury model, depletion of platelets or pretreatment with antibodies to PDGF attenuated neointimal formation.^12^, ^13^ These studies demonstrated that inhibition of PDGF selectively reduced intimal VSMC migration without affecting early medial VSMC proliferation. In contrast, pretreatment with antibodies to bFGF inhibited early medial proliferation, but not later intimal migration and proliferation of VSMC.^14^ Furthermore, systemic infusion of bFGF increased intimal hyperplasia following balloon injury in rats.^15^ However, infusion of PDGF or bFGF did not induce intimal lesion formation in the absence of injury. Taken together, these observations suggest that injury does more than simply release growth factors. Release of other mediators has been considered as an essential cofactor for intimal cell formation. Indeed, models of balloon injury and vein grafting have documented an elevation of extracellular proteases, including plasminogen activator^16^ and matrix metalloproteinases, following balloon injury or vein grafting.^17^, ^18^, ^19^ Animal models suggest that cytokines and proteases released from activated leukocytes initiate protease secretion and activation, resulting in a switch in SMC phenotype, from a differentiated and contractile phenotype to a proliferative and synthetic state, resulting in adventitial and medial remodeling.

## NONCODING RNA

Our current knowledge of the role of RNA in gene regulation has emerged from recent advances in genomic techniques, allowing us to study full transcriptomes of organisms. These technical advances have revealed that mammals produce thousands of noncoding RNA and evidence suggests that in mammals noncoding RNA constitute a substantial majority of transcripts within the genome. It has been suggested that as much as 98% of the human genome encodes for noncoding transcripts, and speculate that differences in organism complexity may arise from the vast differences in noncoding transcripts between higher and lower organisms.^20^ Many of these noncoding transcripts are processed to generate small noncoding RNA such as miRNA, or lncRNA. Through their interaction with DNA, RNA and proteins noncoding RNA have emerged as key regulators of gene expression under both physiological and pathological conditions, as detailed below. Interestingly, cross‐regulatory networks between miRNAs and lncRNA have recently been identified.^21^ A number of detailed sequencing studies have revealed that lncRNA are preferentially expressed in a tissue‐specific manner, suggesting that they hold great promise as selective targets in disease.^22^ The presence of lncRNA selective expression in cells and tissues highlights a potentially novel therapeutic route for controlling disease pathways.

## MicroRNA BIOSYNTHESIS AND FUNCTION

**Figure 2:**
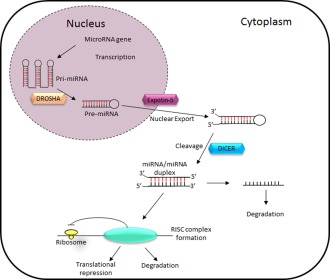
Basic miRNA processing pathway. MiRNA are transcribed from DNA and are cleaved into pre‐miRs via the actions of DROSHA. Pre‐miRs are then exported into the cytoplasm via exportin‐5 and processed into mature miRNA via the actions of DICER. Following their processing the mature miRNA will then associate with a complex called RNA induced silencing complex (RISC) to induce either translational repression or mRNA degradation. Adapted from Ref. 67.

MiRNAs belong to a class of small (18–22 nucleotide, nt), endogenous noncoding RNA molecules that negatively regulate gene expression by targeting specific messenger RNAs, thereby inducing their degradation or translational repression.^23^ MiRNAs are generated via a highly controlled and regulated pathway, as shown in **Figure** 2. This pathway involves two processing events that lead to mature miRNA formation. First, primary miRNAs (pri‐miRNAs) are transcribed by RNA polymerase II and processed in the nucleus through activity of DROSHA into precursor miRNAs (pre‐miRNAs) that are then transported into the cytoplasm, via exportin 5. This double‐stranded 60–90 nt miRNA precursor forms the classical stem and loop structure, as shown in the cytoplasm of **Figure** 2. In the cytoplasm, pre‐miR‐s are cleaved by DICER into a mature RNA duplex that associates with argonaute proteins that load specific miRNA strands into the RNA‐induced silencing complex^24^ to form a RISC‐miRNA complex that subsequently represses mRNA transcription or enhances mRNA degradation.

## lncRNA BIOSYNTHESIS AND FUNCTION

**Figure 3:**
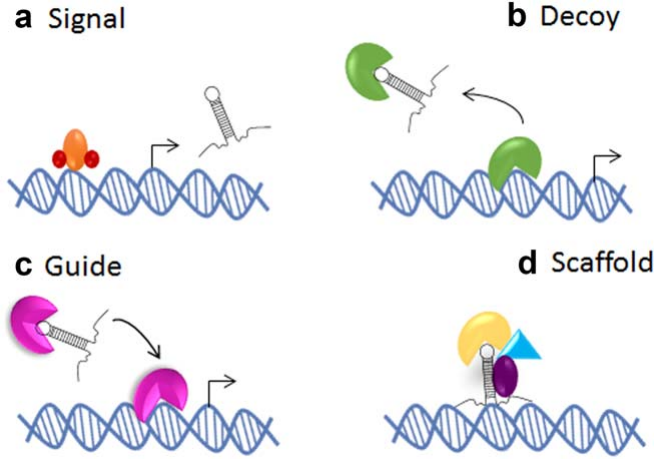
Schematic diagram of four archetypes of lncRNA mechanisms. lncRNA can exert their function through complementary binding to RNA, DNA, and protein molecules. Adapted from Ref. 26. (**a**) lncRNA may act as signals of transcriptional activity and may indicate gene regulation. (**b**) lncRNA may act as endogenous sponges for molecules such as miRNA, thus reducing the bioavailability of the molecule, altering cellular function. (**c**) lncRNAs can act as guides and tethers for chromatin‐modifying complexes, thus aiding in the recruitment to DNA and contributing to tissue‐specific gene expression. (**d**) lncRNA may act as scaffolds bridging essential proteins required for gene or cellular regulation.

lncRNAs are a large family of transcribed RNA molecules with a length of more than 200 nt with little or no known protein coding potential. lncRNAs are transcribed throughout the genome and display remarkable similarity to classical mRNA in that they are translated by RNA polymerase II and are generally, but not always, alternatively spliced and polyadenylated.^25^ They are highly versatile and function to regulate gene expression by diverse mechanisms. lncRNA can partially basepair with DNA or RNA in a sequence‐specific manner, or form complexes with proteins. Recent attempts have been made to categorize the various types of molecular mechanisms involved in lncRNA function. As such, lncRNA may be defined by four different archetypes (**Figure** 3). Archetype 1: signal archetype meaning that the lncRNA functions as a molecular signal or indicators of transcriptional activity. Archetype 2: Decoy archetype: lncRNA can bind to and titrate away other regulatory RNA or proteins. Achetype 3: Guide archetype: lncRNA direct the localization of specific ribonucleotide proteins to their specific targets. Finally, archetype 4: Scaffold archetype: lncRNA can act as a structural platform upon which relevant components may act to stabilize nuclear structures or signaling complexes.^26^

## MECHANISM OF lncRNA‐MiRNA INTERACTIONS

Recent studies have identified that lncRNA can bind to miRNA in order to “communicate” with other RNA targets.^27^ An abundance of lncRNA have been shown to be regulated by miRNA and the reciprocal. Since many of these control important physiological functions, the abundance and binding of each miRNA and lncRNA will directly alter cellular function.

**Figure 4:**
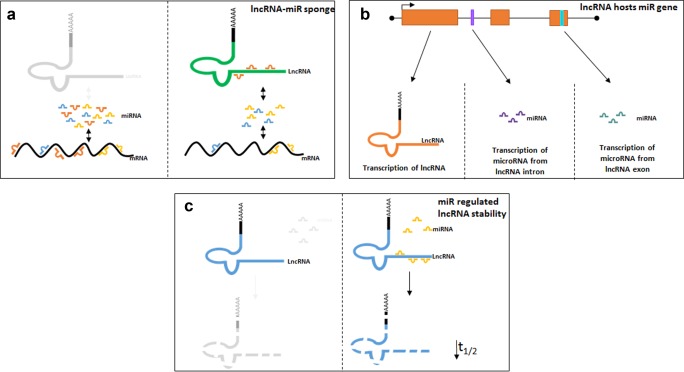
lncRNA: miRNA‐mediated interactions. Schematic diagram highlighting the routes via which lncRNA and miRNA may interact. (**a**) lncRNA‐miRNA sponge: binds and sequesters miRNA away from their site of action, thus reducing their function within the cell. (**b**) lncRNA generating miRNA. lncRNA may host miRNA both within their exons and introns. (**c**) Some lncRNA are degraded by miRNA.

**Figure 5:**
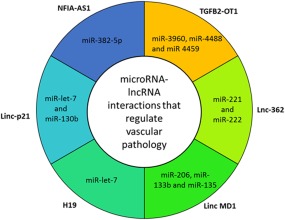
Overview of lncRNA‐mediated interactions in vascular smooth muscle and endothelial cells during vascular disease. Many novel lncRNA have been identified to bind and regulated miRNA function. Outside labels indicate the lncRNA involved in the interaction. Inner ring highlights the miRNA associated with the interacting lncRNA.

By sharing common miRNA binding sites with mRNA targets, lncRNA can sequester and compete with miRNA to prevent miRNA function and alleviate mRNA repression (**Figure** 4 **a**).^28^ Interestingly, ∼40% of miRNA are found in introns of protein coding genes.^29^ Subsequent analysis has shown that ∼10% of lncRNA genes also host an miRNA, either in an intron or exon^30^ (**Figure** 4 **b**). Finally, miRNAs have been shown to bind to lncRNA, with the addition of other RNA binding proteins, to regulate lncRNA stability and miRNA‐mediated decay (**Figure** 4 **c**). In this review, we describe and discuss lncRNA‐miRNA networks in both endothelial and smooth muscle cells during vascular disease. An overview of novel lncRNA and miRNA interactions are shown in **Figure** 5.

## TGFβ2‐OT1 AND MiR‐3960, MiR‐4488, AND MiR‐4459

Transforming growth factor beta 2 overlapping transcript 1 (TGFβ2‐OT1) is a recently identified lncRNA derived from the 3′UTR (untranslated region) of TGFβ2. TGFβ2‐OT1 expression is regulated through lipopolysaccharide^31^ and oxidized low‐density lipoprotein, the central mediator involved in the initiation and progression of atherosclerosis. Microarray profiling following TGFβ2‐OT1 overexpression revealed that TGFβ2‐OT1 regulated the expression of three miRNAs (miR‐3960, miR‐4488, and miR‐4459). Subsequent interrogation revealed that TGFβ2‐OT1 acts as an endogenous competing RNA bound to miRNAs (**Figure** 3 **a**). Furthermore, overexpression of miR‐3960, miR‐4488, and miR‐4459 resulted in a repression of their downstream targets CERS1 (ceramide synthase 1), NAT8L (N‐acetyltransferase 8‐like), and LARP1 (La ribonucleoprotein domain family, member 1).^32^ Intriguingly, all three targets are involved in endothelial cell autophagy and inflammation, central processes involved in endothelial injury.

In addition, an increase in miR‐3960 expression is associated with areas of increased arterial calcification, which is a hallmark of advanced atherosclerosis and plaque stability. Intriguingly, both TGFβ and bone morphogenic protein (BMP) play a fundamental role in vascular remodeling and calcification. Activation of these signaling molecules triggers several diverse downstream pathways, including activation of the RUNX2 transcription factor, an essential and sufficient regulator of vascular calcification.^33^ Furthermore, MiR‐3960 expression has been shown to be induced by activation of this osteogenic transcription factor. Additionally, overexpression of miR‐3960 elevated the expression of the osteoblast differentiation markers alkaline phosphatase (ALP), osteocalcin, and RUNX2, thus promoting vascular calcification.^34^ Taken together, these results suggest that overexpression of TGFβ2‐OT1, and subsequent downregulation of miR‐3960, may prove a novel way to specifically target RUNX2 signaling in the setting of atherosclerosis and vascular calcification.

## Lnc362 AND MiR‐221 AND MiR‐222

Aberrant regulation of angiotensin II (Ang II) has been linked to hypertension and atherosclerosis.^35^ Following RNA‐sequencing in Ang II‐treated cells, Leung *et al*. identified a collection of Ang II‐responsive lncRNA. Among these, lncRNA 362 was identified.^36^ lncRNA 362 is located proximal to two miRNA, miR‐221 and miR‐222, which have been linked with regulation of SMC proliferation via suppression of c‐kit and p27kip.^37^, ^38^ Knockdown of lncRNA 362 is associated with a reduction in SMC proliferation and expression levels of these miRNAs, indicating that these miRNAs are coregulated with lncRNA‐362. Furthermore, specific manipulation of either miRNA has been shown to reduce neointimal formation associated with smooth muscle cell proliferation.^37^ In accordance with this study, Bazan *et al*. identified that miR‐221 and miR‐222 were significantly downregulated in the shoulder region of atherosclerotic plaques. Atherosclerotic plaque vulnerability is accompanied by changes in the molecular and cellular function in the plaque shoulder, including a decrease in VSMC proliferation. This reduction in miR‐221/miR‐222 promotes the formation of an unstable plaque via decreased p27Kip1 mRNA expression, as described above, encouraging plaque rupture.^39^

Atherosclerosis initiation is linked to vascular endothelial injury and subsequent recruitment of leukocytes to activated endothelial cells.^40^ It has been previously demonstrated that miR‐221/222 can inhibit tube formation, migration, and wound healing by directly targeting c‐Kit. Zhu *et al*. have shown overexpression of miR‐221/222 in HUVECs downregulates key adhesion molecules on vascular endothelial cells, thus altering inflammatory pathways involved in atherosclerosis initiation.^41^

These studies raise the intriguing possibility that modulation of lncRNA could provide a novel mechanism to attenuate the hyperproliferative effects of Ang II within multiple vascular pathologies, including coronary artery disease, PAH, and atherosclerosis since both these miRNAs are implicated in proliferative vascular remodeling.

## Linc MD1 AND MiR‐206, MiR‐133B, AND MiR‐135

Unlike miRNA, lncRNA are not confined to a single mechanism of action. One example of this is Linc‐MD1, a muscle‐specific lncRNA with two distinct mechanisms of action for controlling physiology. As shown in **Figure** 4 **b**, some lncRNA are processed to generate miRNA. Linc‐MD1 generates miR‐206, in one intron, and miR‐133b, in one exon.^42^ MiR‐206 has previously been linked to vascular remodeling during pulmonary arterial hypertension. These studies demonstrated that miR‐206 expression is associated with increased right ventricular systolic pressure.^43^ Reduction in levels of miR‐206 resulted in increased levels of smooth muscle cell differentiation markers, α‐smooth muscle actin and calponin, implicating its importance in the differentiation of SMCs. In addition, miR‐206 overexpression results in a composite downregulated of Notch 3 expression, a key mediator in PAH development. Taken together, these studies suggest that miR‐206 regulates vascular remodeling in the setting of pulmonary hypertension via a number of different mechanisms, which play a prominent role in disease pathology (smooth muscle cell proliferation, apoptosis, and differentiation), suggesting that it may have an enhanced therapeutic efficacy of pulmonary arterial SMC.

Although no direct link between PAH and miR‐133b has been established in the lung, miR‐133b has been implicated as a biomarker of right ventricle hypertrophy and therefore a biomarker of PAH.^44^

Additionally, Linc‐MD1 also contains binding sites for miR‐135 and miR‐133, thus Linc‐MD1 can not only produce miR‐133 but can also control its function via acting as a sponge for its own produced miRNA. Unlike lncRNA that regulate gene expression at the epigenetic level, Linc‐MD1 is located solely in the cytoplasm of cells. In addition to its role in pulmonary arterial hypertension, miR‐133b has been shown to be involved in vascular calcification. *In vitro* and *in vivo* vascular calcification models identified miR‐133b as being significantly downregulated, while miR‐133b target RUNX2, as mentioned above, was significantly elevated. Additionally, the expression of several inhibitors of vascular calcification were reduced in both *in vitro* and *in vivo* models.^45^ In accordance with this study, Cipollone *et al*. showed an increase in miR‐133 expression in symptomatic compared to asymptomatic carotid plaques.^46^ Thus, modulation of Linc‐MD1 may prove beneficial in the pathogenesis of other vascular pathologies, such as atherosclerosis.

## H19 AND MiR‐LET‐7

H19 is an lncRNA conserved across humans and rodents. Abundantly expressed in human muscle, the aberrantly expressed lncRNA, H19, has been implicated in several genetic conditions.^47^ Recently, the functional role of H19 had remained unknown, until Kallen *et al*. discovered that H19 harbors both canonical and noncanonical binding sites for the let‐7 family of miRNAs.^48^ Utilizing both knockdown and overexpression to modulate H19 expression, the authors discovered that H19 modulates let‐7 availability by acting as a “molecular sponge,” reducing the level of free let‐7 able to bind its target mRNA. Physiologically, this has implications in the control of the SMC phenotype, where knockdown of H19 caused precocious muscle differentiation, a phenotype recapitulated by let‐7 overexpression.

Additionally, the role of lncRNA H19 has been assessed in rats *in vivo*. H19 was undetectable in uninjured carotid arteries; however, 7 and 14 days postinjury, H19 was abundantly expressed and localized to the neointimal area following *in situ* analysis. Intriguingly, H19 was initially identified as a developmental lncRNA, known to be expressed during development and downregulated after birth.^49^ This highlights the notion that the vascular response to injury is accompanied by the reexpression of several fetal gene networks.^50^

H19 lncRNA is a primary precursor for miR‐675, located in the first exon. Although known to act as an effective biomarker in heart failure patients,^51^ and to reduce proliferation of a range of embryonic cell lines,^52^ no link between miR‐675 and vascular endothelial or smooth muscle cells has been identified. This may provide an interesting avenue for further investigation.

## Linc‐P21 AND MiR‐LET‐7 AND MiR‐130b

Unlike miRNA, lncRNA are not as well conserved. A recent report suggests that up to 44% of lncRNA may be conserved between mammals^22^; however, others show the number to be much lower.^53^ One such conserved lncRNA is LincRNA‐p21, a novel lncRNA located ∼15kb upstream of the cell cycle regulator gene p21. Linc‐p21 is ∼3 kbp in length and has been shown to promote cell proliferation, apoptosis, and DNA damage response in a variety of disease states.^54^

Unlike the more conserved miRNA, lncRNA can have multiple mechanisms of action. One example of this is the long noncoding RNA Linc‐p21. Linc‐p21 is a transcriptional target of p53, a tumor suppressor that regulates cell cycle. The expression of Linc‐p21 is dramatically downregulated in atherosclerotic lesion from mice maintained on an atherogenic background. Subsequent gain‐ and loss‐of‐function studies revealed that loss of Linc‐p21 expression is associated with an increase in SMC proliferation and reduction in apoptosis in vascular smooth muscle cells. Furthermore, lentiviral‐mediated knockdown of Linc‐p21 expression enhanced neointimal formation in murine arteries subjected to wire injury, events associated with increased SMC proliferation and survival. Genome‐wide analysis revealed that LincRNA‐p21 inhibition dysregulated many p53 targets. This study was one of the first to demonstrate that modulation of lncRNA has therapeutic potential in the setting of acute vascular injury.

In addition to lncRNA controlling miRNA expression and function, miRNA can also regulate lncRNA expression via binding and altering lncRNA function. Recently, PAR‐CLIP (photoactivatable ribonucleoside‐enhanced crosslinking and immunoprecipitation) analysis revealed that the RNA binding protein (RBP) HuR associates with several lncRNAs. HuR is a ubiquitous RBP that induces cellular proliferation, apoptosis, and cellular immune responses. HuR performs most of these activities via binding and regulating target RNA stability. Interestingly, Yoon *et al*. identified that binding of both HuR and miRNA let‐7 decreased Linc‐p21 stability, leading to its degradation and loss of function, an effect common with miRNA on mRNA^55^ (**Figure** 4 **c**). Additionally, linc‐p21 has also been shown to act as an endogenous sponge to miRNA‐130b in both vascular endothelial and smooth muscle cells. MiR‐130b has been implicated in both endothelial cell and smooth muscle cell pathology. In endothelial cells, miR‐130 modulated apelin‐miR‐424/503‐FGF2 signaling, while in smooth muscle cells miR‐130 modulated STAT3‐miR‐204 signaling to promote PAH‐associated phenotypes.^56^

## NFIA‐AS1 AND MiR‐382‐5p

As mentioned previously, smooth muscle and endothelial cells are not the only cell types to play a prominent role in vascular disease. Via microarray techniques, Hu *et al*.^57^ discovered that lncRNA NFIA‐AS1 expression was upregulated, whereas nuclear factor IA (NFIA) expression was downregulated in a human THP‐1 foam cell model. As it has been previously shown that lncRNA can affect target genes, the authors assessed known NFIA‐regulated miRNA. The data suggest that NFIA‐AS1 does not bind directly to miR‐382‐5p but acts as an enhancer via some additional mechanism to promote the expression of this miRNA. These data were also confirmed through subsequent knockdown of this lncRNA *in vitro*. In a mouse model, lentiviral‐mediated overexpression of this lncRNA affected central mechanisms of atherogeneisis such as the ratio of high‐density lipoprotein (HDL) to low‐density lipoprotein (LDL) in the circulation, increased HDL cholesterol, reduced LDL cholesterol, and decreased circulation of inflammatory cytokines, IL‐1β, IL‐6, TNF‐α, and C‐reactive protein (CRP). Overexpression also reduced atherosclerosis in apolipoprotein E‐deficient (*Apoe–/–*) mice.^57^ In humans the NFIA gene locus has not been associated with atherosclerosis to date, nor has miR‐382‐5p been investigated in atherosclerosis or any other vascular disease. However, this article highlights lncRNA as master regulators of disease and may highlight NFIA‐AS1 as an important target for atherosclerosis treatment.

## CLINICAL UTILITY OF lncRNA

### lncRNA as diagnostic biomarkers

For clinical medicine, lncRNA offer many benefits. lncRNA typically show tissue‐restricted and vascular disease‐restricted patterns of expression.^58^, ^59^ Given this specificity, lncRNA may be superior biomarkers than current protein coding genes for cardiovascular disease. Additionally, lncRNA in themselves are functional molecules, thus their expression may be a better indicator of disease states.^60^

One prominent example is CoroMarker (AC100865.1), a lncRNA that is markedly overexpressed in coronary artery disease (CAD). The authors identified CAD biomarkers using the strict criteria of signal intensity ≥8, fold change >2.5, and *P* < 0.005. The predictive value of CoroMarker was assessed in a large cohort with 221 CAD patients and 187 control individuals. CoroMarker was able to successfully identify 78.05% of CAD patients.^61^ CoroMarker is stably expressed within the plasma and further tests to evaluate its ability to detect CAD are currently underway.

### lncRNA as therapeutics

The transition from lncRNA biomarkers to lncRNA therapeutics is also showing promising advances. Companies and organizations such as Miragen Therapeutics and Regulus are developing ncRNA‐based strategies against cancer, cardiovascular, and neurological diseases. Although lncRNA clinical trials are still several years away, it is generally accepted that targeting of lncRNA therapeutically may lead to less off‐target effects, due to their enhanced tissue specificity and their ability to modulate miRNA/mRNA networks. Additionally, the advantage of using RNA as a therapeutic medium would allow quick regulatory functions to be altered without the need for protein translation. One such noncoding RNA therapeutic, miravirsen, is currently in phase II clinical trials for the treatment of hepatitis C. Miravirsen is an inhibitor of miR‐122, a liver‐specific miRNA that the hepatitis C virus requires for replication. Miravirsen is designed to sequester miR‐122, making it unavailable to the hepatitis C virus.^62^ This highlights one mechanism by which noncoding RNA can be modulated; however, there are several other therapeutic approaches for targeting lncRNA in man:

1. Therapeutic silencing of lncRNA. lncRNA expression can be altered through the use of RNAi technology. Therapeutic RNAi technology has now been tested in several mammals including man.^63^, ^64^ Current clinical trials are investigating the safety and efficacy of RNAi‐mediated therapeutics and it could be relatively easily adapted to allow RNAi therapies targeting lncRNA.
2. Functional block of lncRNA. This could be achieved through the utilization of small molecules that block binding sites on protein interacting partners or oligonucleotides that bind to the ncRNA, blocking the interaction with partners such as miRNA, while allowing the lncRNA to bind additional partners at other sites not associated with disease. Current research has already assessed the use of high‐throughput screening to identify small molecule disruptors for the lncRNA HOTAIR.^65^
3. Structure disruption. Small molecules can be utilized to bind to the lncRNA of interest and disrupt its secondary structure, thus disrupting the binding site on the lncRNA or small molecules can be utilized to mimic the structure of the lncRNA, thus competing with the lncRNA for target sites. Research is underway to achieve this goal.^60^, ^66^
4. Finally, gene therapy could be utilized to deliver beneficial lncRNA.

Due to their multiple functional roles, the therapeutic potential of lncRNA is extremely vast. Ultimately, further analysis of lncRNA structure and functional roles will be expected before these therapeutic options can be used routinely.

## CONCLUSION

Exploring the transcriptome that controls SMC and EC phenotype is of key importance in unraveling the regulatory pathways and disease mechanisms of vascular pathology. Greater understanding of the complex interplay between RNA and protein coding genes will undoubtedly present new therapeutic applications and diagnostic implications in vascular pathology.

## CONFLICT OF INTEREST

The authors declare no conflicts of interest.
